# RyhB Regulates Capsular Synthesis for Serum Resistance and Virulence of Avian Pathogenic *Escherichia coli*

**DOI:** 10.3390/ijms26073062

**Published:** 2025-03-27

**Authors:** Yuxing Shi, Mingjuan Gao, Lin Xing, Guoqiang Zhu, Heng Wang, Xia Meng

**Affiliations:** 1 Jiangsu Co-Innovation Center for the Prevention and Control of Important Animal Infectious Diseases and Zoonoses, College of Veterinary Medicine, Yangzhou University, Yangzhou 225009, China; syx17806130969@163.com (Y.S.); gao41724@163.com (M.G.); 13291902836@163.com (L.X.); yzgqzhu@yzu.edu.cn (G.Z.); wh@yzu.edu.cn (H.W.); 2 Joint International Research Laboratory of Prevention and Control of Important Animal infectious Diseases and Zoonotic Diseases of China, Yangzhou 225009, China

**Keywords:** RyhB, APEC, capsular synthesis, serum resistance, virulence regulation

## Abstract

Avian pathogenic *Escherichia coli* (APEC) causes bloodstream infections mainly by resisting the bactericidal action of host serum. Although various protein and polysaccharide factors involved in serum resistance have been identified, the role of small non-coding RNA (sRNA) in serum resistance has rarely been studied. The sRNA RyhB contributes to serum resistance in APEC, but the regulation mechanism of RyhB to serum resistance-related targets remains unknown. Here, we studied the regulatory mechanism of RyhB on capsule synthesis and how RyhB regulates serum resistance, macrophage phagocytosis resistance, and pathogenicity to natural hosts by regulating capsule synthesis. The results showed that RyhB upregulates capsular synthesis by interacting with the promoter regions of the capsule gene cluster and activating the translation of the capsule. The deletion of *ryhB* and/or *neu* reduced the ability of resistance to serum, macrophage phagocytosis, and pathogenicity of APEC in ducks. It can be concluded that RyhB directly upregulates the expression of capsular gene cluster and capsular synthesis and then indirectly promotes resistance to serum and macrophage phagocytosis and pathogenicity to ducks.

## 1. Introduction

Avian pathogenic *Escherichia coli* (APEC) is an important member of extraintestinal pathogenic *Escherichia coli* (ExPEC) that causes disease outside the gut such as bacteremia, pericarditis, and meningitis in poultry [1]. APEC strains that cause meningitis mainly belong to the B2 phylogenetic group with the dominant serotypes O18, O2, and O1 [2,3]. The strain APEC XM (O2:K1:H7) used in our study also belongs to group B2 and causes severe bacteremia and meningitis in ducks and mice [4,5]. Duck is the second most predominant poultry in Chinese poultry farming. A large number of APECs were isolated and caused colibacillosis in duck, resulting in economic losses in the duck industry [6,7]. APEC employs similar pathogenic strategies with neonatal meningitis *Escherichia coli* (NMEC) in causing meningitis and shows a potential zoonotic risk [2]. The meningitis APEC strains have the ability to cause bloodstream infections, leading to bacteremia or septicemia. Adaption to bloodstream environment and serum resistance to serum damage are important for bloodstream infections. Numerous factors such as K-capsule [8,9], O antigen [10], lipopolysaccharide [11], and outer membrane protein OmpA [12] are reported to contribute to the serum resistance of ExPEC. Although polysaccharide and proteins have been proved to play an important role in serum resistance, the effect of small non-coding RNA (sRNA) in serum resistance has not been revealed in APEC.

Bacterial trans-encoded sRNAs are a class of RNAs that regulate the expression of genes mainly by an incomplete complementary base pairing mechanism [13,14]. RyhB is a trans-encoded sRNA with a size of about 90 nucleotides in bacteria [15]. RyhB regulates multiple physiological processes of bacteria, including iron homeostasis [15], oxidative stress [16], nitrate metabolism [17], and acid resistance [18]. What is more noteworthy is that RyhB contributes to the pathogenicity of pathogens by upregulating virulence-related characteristics such as adhesion to epithelial cells [4], biofilm formation [19], and survival in macrophages [20]. Our previous study revealed that RyhB in APEC could respond to serum and hypoxic stress environments and contribute to the survival in serum [4]. Moreover, RyhB increased the bacterial load of APEC in blood, brain, and spleen, enhanced the damage of APEC to the blood–brain barrier of mice, and contributed to the occurrence of meningitis in mice [4]. However, the target genes regulated by RyhB and the regulation mechanism of RyhB in APEC pathogenicity are unclear.

As a steric barrier of ExPEC, the capsule is the critical determinant in serum resistance [21] and the development of *E. coli* meningitis in the rat [22]. The capsule gene clusters (*kps*) of group B2 *E. coli* are composed of three regions [23]. Region 1 genes (*kpsFEDUCS*) and Region 3 genes (*kpsMT*) are conserved in group B2 *E. coli* and encode proteins responsible for transporting capsular polysaccharides from the cytoplasm to the cell surface. Region 2 genes (*neuDBACES*) are specific in different serotypes and encode proteins for the synthesis of capsular polysaccharides and their precursors [23]. The expression of the capsule gene cluster is regulated by two temperature-regulated promoters, PR1 and PR3 [24]. PR1 is a 225 bp length sequence upstream of *kpsF*, while PR3 is a 741 bp length fragment upstream of *kpsM*, which is a shared promoter of Regions 2 and 3. A global regulator, histone-like nucleoid structuring protein (H-NS), and its anti-repressor, SlyA, regulate the *kps* cluster by binding sites in the promoter PR1 and PR3 [25,26]. A global regulator Integration Host Factor (IHF) [27] and the transcriptional anti-terminator RfaH [28] also control the expression of the K-capsule by directly binding to the PR1 and PR3 promoters, respectively. In our previous study, we found that the deletion of *ryhB* decreased the expression of all genes in the *kps* significantly [4]. It is supposed that RyhB regulates the expression of capsule and affects the virulence of APEC. Here, we aimed to elucidate the regulatory mechanism of RyhB on the capsular genes and further clarify whether RyhB affects the virulence of APEC by regulating the capsular synthesis, resistance to serum, and macrophage phagocytosis.

## 2. Results

### 2.1. Deletion of ryhB Affects the Expression of Capsule Synthesis-Associated Genes

**Figure 1:**
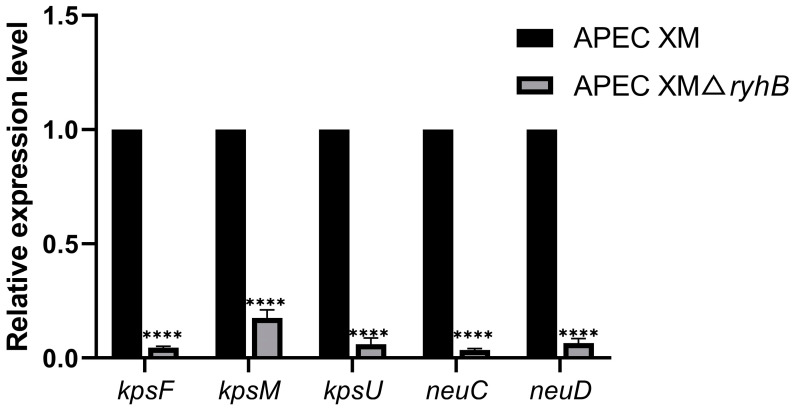
Relative expression levels of capsule synthesis-associated genes in APEC XMΔ*ryhB* compared with those in WT. The expression levels of the above genes in WT were used as the baseline, and the value is defined as 1. The data are represented as mean ± standard deviations (SDs) from triplicate independent experiments. Statistical analysis was performed using unpaired Student’s *t*-test for independent samples by GraphPad Prism software. Significant differences were indicated by *p*-values. A *p*-value of less than 0.05 was considered statistically significant. **** *p* < 0.0001.

To determine if RyhB affects the capsule synthesis when APEC survived in duck serum, the transcriptional levels of some capsule synthesis-associated genes that contained *kpsF*, *kpsM*, *kpsU*, *neuC*, and *neuD* were detected by quantitative real-time PCR (qRT-PCR) (Figure 1). The expressions of all of these genes were decreased in the APEC XMΔ*ryhB* mutant compared with that in the wild type (WT) strain. In particular, the expressions of *neuC* and *neuD* genes that are responsible for polysaccharide synthesis were decreased by more than 20-fold in the APEC XMΔ*ryhB* mutant (*p* < 0.0001). *kpsF* and *kpsU*, Region 1 genes of capsule gene cluster, were also significantly decreased compared with those in the WT strain. The result indicated that RyhB could upregulate the expression of the capsular genes when APEC was challenged with serum.

### 2.2. RyhB Directly Upregulates Capsular Gene Clusters by Interacting with the 5′ UTR of kpsF and kpsM

**Figure 2:**
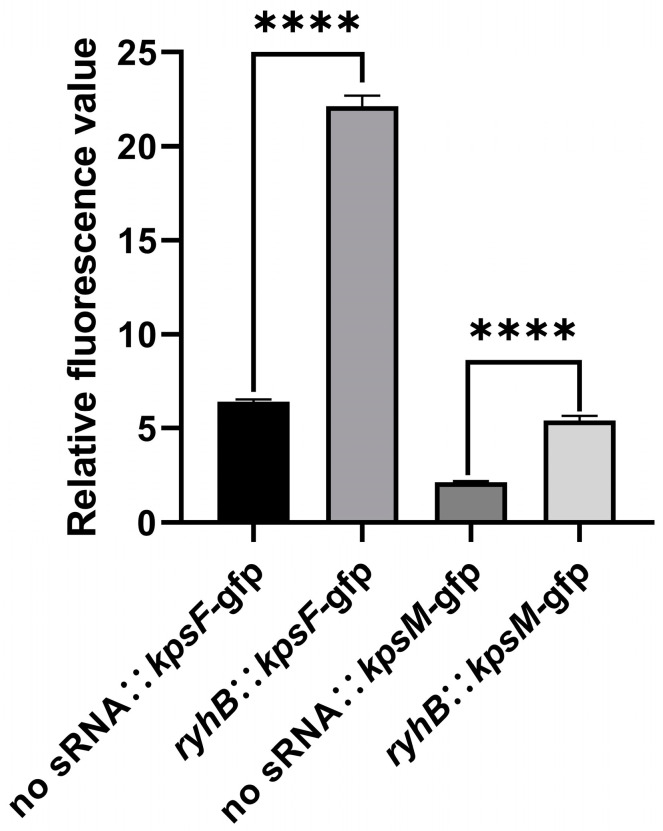
Relative fluorescence values of bacteria when cultured in LB liquid medium. The data are shown as mean ± SDs from triplicate experiments. Statistical analysis was performed using unpaired Student’s *t*-test by GraphPad Prism software, and significant differences were indicated by *p*-values. **** *p* < 0.0001.

The result of RyhB and the 5′ untranslated region (UTR) of capsular gene clusters interaction site prediction showed that a region (nt 70–88) in RyhB could form base pairs with the 5′ UTR of *kpsF* (nt 135–152), while a region (nt 50–86) in RyhB could form base pairs with the 5′ UTR of *kpsM* (nt 32–75) (Figure S1). The secondary structure of the 5′ UTR and the first 150 bases of the *kpsF* mRNA were predicted using RNAstructure. The result indicated that the sequence of 135th to 152nd nt 5′ UTR of *kpsF* formed a stem-loop structure. The sequence of 30th to 86th nt 5′ UTR of the *kpsM* mRNA was also predicted to form a long stem-loop structure (Figure S1). A stem-loop structure may block the binding of ribosomes to the Shine–Dalgarno (SD) sequence and decrease the translation efficiency of the whole capsular gene cluster. Based on the results of interaction site prediction, we hypothesize that RyhB binds to the 5′ UTR of *kpsF* and *kpsM*, prevents the stem-loop structure’s formation, and then promotes the translation of proteins in the capsular operon. The GFP-based reporter system was used to determine the relative fluorescence intensity of strains containing *ryhB* and 5′ UTR of mRNA expression plasmids. The result showed that the relative fluorescence intensity of the strain carrying “*ryhB*:: *kpsF*-*gfp*” was significantly stronger than the strain carrying “no sRNA:: *kpsF*-*gfp*” (Figure 2). This indicated that RyhB could directly upregulate the expression of *kpsF*, and probably even the expression of the region of *kpsFEDUCS* by interacting with the 5′ UTR of *kpsF*. The same method also determined that RyhB could upregulate the expression of *kpsM*, and probably even the expression of the region of *kpsMT* and *neuDBACES*.

### 2.3. RyhB Contributes to the Production of Capsules

**Figure 3:**
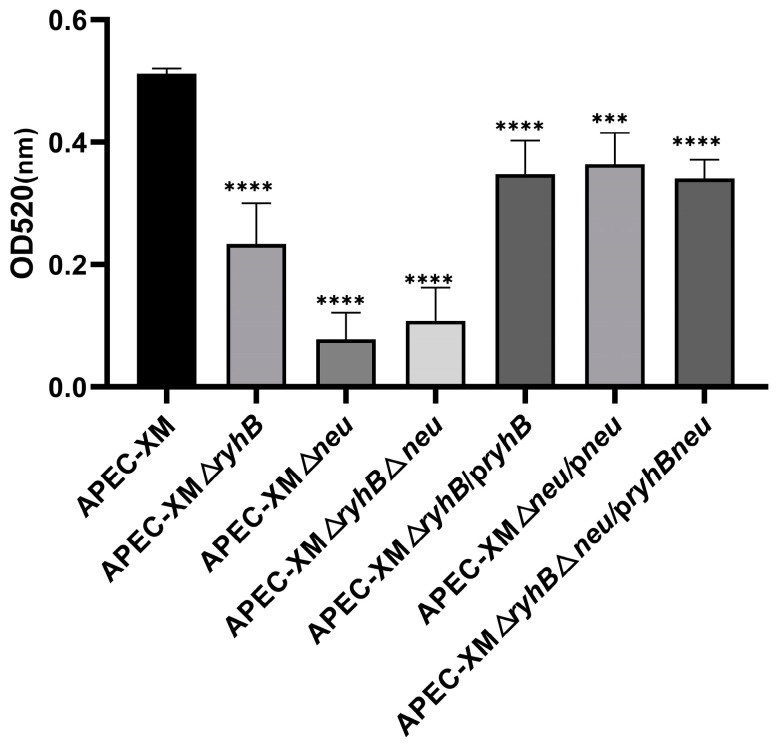
The production of extracellular polysaccharides of bacteria in serum under low-oxygen condition after 8 h incubation. The absorbance at 520 nm was measured to quantify the production. The data are shown as mean ± SDs from three independent experiments. The differences between APEC XM and the mutants were statistically analyzed using one-way analysis of variance (ANOVA) followed by Tukey’s HSD test for multiple comparisons, performed using GraphPad Prism software. Significant differences were indicated by *p*-values. *** *p* < 0.001, **** *p* < 0.0001.

To determine whether the deletion of *ryhB* affects capsules production, the extracellular polysaccharides in APEC (mainly contain K capsules and colonic acids) were extracted and quantified under serum and low-oxygen conditions. The APEC XMΔ*neu* mutant which lacked capsular synthesis genes *neuDBACES* showed a most significant decrease in capsules production. The capsules production of the APEC XMΔ*ryhB* mutant also decreased, but less than that of the APEC XMΔ*neu* mutant (Figure 3). This indicates that the deletion of *ryhB* affects the capsule synthesis to a certain extent but cannot completely affect the synthesis ability of the capsule. In other words, RyhB contributes to capsule synthesis. The production of the capsule in the double deletion mutant APEC XMΔ*ryhB*Δ*neu* mutant is similar to that in the APEC XMΔ*neu* mutant, but less than that of the APEC XMΔ*ryhB* mutant without a significant difference. All the complemented mutants APEC XMΔ*ryhB*/p*ryhB*, APEC XMΔ*neu*/p*neu*, and APEC XMΔ*ryhB*Δ*neu*/p*ryhBneu* partly restored the ability of capsule synthesis.

### 2.4. RyhB Is Required for Serum Resistance of APEC

The assay of survival of the APEC XM, the deletion mutants APEC XMΔ*ryhB*, APEC XMΔ*neu*, and APEC XMΔ*ryhB*Δ*neu*, and the complemented mutants APEC XMΔ*ryhB*/p*ryhB*, APEC XMΔ*neu*/p*neu*, and APEC XMΔ*ryhB*Δ*neu*/p*ryhBneu* in duck serum showed that the survival rates of all of the deletion mutants were declined, while that of APEC XM first decreased and then increased from 1 h to 2 h. The survival of all the complemented mutants was restored. This indicated that the deletion of *ryhB* and/or *neu* led to a decrease in the survival ability of APEC.

### 2.5. RyhB Promotes Resistance to Phagocytosis

**Figure 4:**
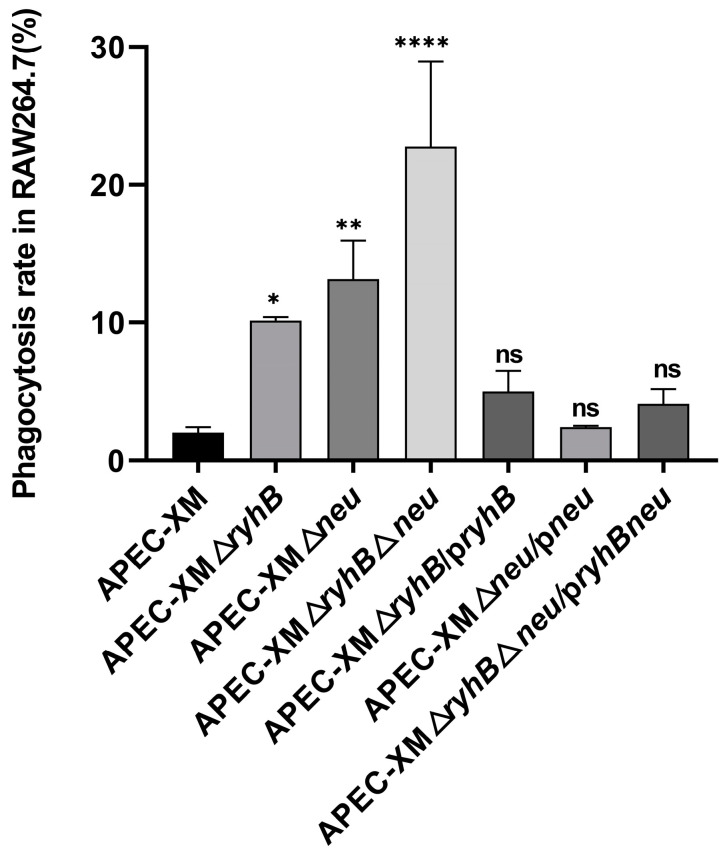
The phagocytosis rate of APEC in RAW264.7 cells. The phagocytosis rate was determined by calculating the bacteria number inside the macrophage to the number of initial bacteria that incubated with the cells. The data are presented as mean ± SDs from triplicate experiments and statistically analyzed using one-way ANOVA by GraphPad Prism software. Tukey’s HSD test was used for multiple comparisons to determine differences between the APEC XM group and the mutant groups. Significant differences were indicated by *p*-values. **** *p* < 0.0001; ** *p* < 0.01; * *p* < 0.05; ns: no significance, *p* > 0.05.

Capsules play an important role in resistance to macrophage phagocytosis. The phagocytosis rates of APEC XM, deletion mutants APEC XMΔ*ryhB*, APEC XMΔ*neu*, and APEC XMΔ*ryhB*Δ*neu*, and complemented mutants APEC XMΔ*ryhB*/p*ryhB*, APEC XMΔ*neu*/p*neu*, and APEC XMΔ*ryhB*Δ*neu*/p*ryhBneu* in RAW264.7 cells were detected to evaluate the function of RyhB in phagocytosis resistance by regulating capsular synthesis. Our result showed that the phagocytosis rate of WT strain APEC XM was 2%, which was the lowest among the tested strains. The phagocytosis rates of the three deletion mutants were significantly higher than those of the WT group, which were 10.13%, 13.16%, and 23%, respectively (Figure 4). This indicated that the deletion of *ryhB* and/or *neuDBACES* reduced the ability of APEC XM to resist macrophage phagocytosis. Compared with APEC XMΔ*ryhB*, APEC XMΔ*neu* had a higher phagocytosis rate, indicating that RyhB could promote resistance to macrophage phagocytosis by upregulating capsule synthesis. The phagocytosis rates of complemented mutants APEC XMΔ*ryhB*/p*ryhB*, APEC XMΔ*neu*/p*neu*, and APEC XMΔ*ryhB*Δ*neu*/p*ryhBneu* were 5.01%, 2.42%, and 4.00%, respectively, which partly restored the ability of resistance to macrophage phagocytosis.

### 2.6. RyhB and Capsule Enhance Virulence of APEC in Ducks

**Figure 5:**
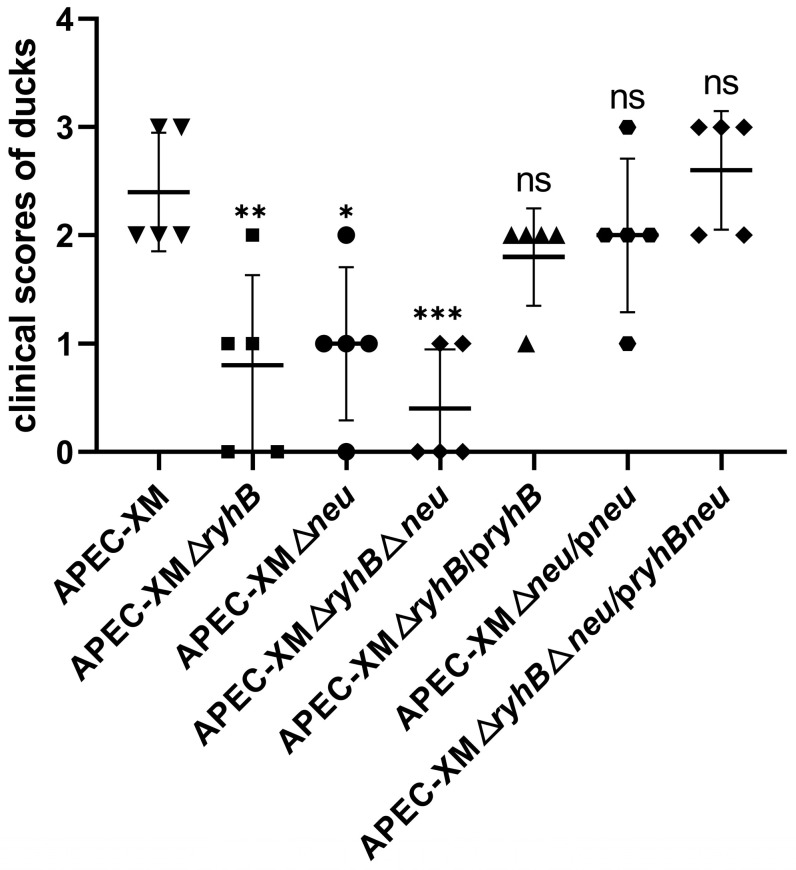
Clinical scores of ducks that were challenged with bacteria. The data are presented as mean ± SDs from five ducks of each group and statistically analyzed using one-way ANOVA by GraphPad Prism software. Tukey’s HSD test was used for multiple comparisons to determine differences between the APEC XM infection group and the mutant infection groups. Significant differences were indicated by *p*-values *** *p* < 0.001; ** *p* <0.01; * *p* < 0.05; ns: no significance, *p* > 0.05. The scatter plot uses different symbols to represent the clinical score of every duck in different groups.

To determine whether RyhB affects the pathogenicity of APEC in ducks by regulating capsular synthesis, ducks were inoculated intraperitoneally with WT strain APEC XM, deletion mutants APEC XMΔ*ryhB*, APEC XMΔ*neu*, and APEC XMΔ*ryhB*Δ*neu*, and complemented mutants APEC XMΔ*ryhB*/p*ryhB*, APEC XMΔ*neu*/p*neu*, and APEC XMΔ*ryhB*Δ*neu*/p*ryhBneu*, respectively. The health status of the ducks was evaluated by a clinical score (Figure 5). The ducks that were challenged with the WT strain exhibited the most serious clinical symptoms (such as diarrhea, depression, and opisthotonus) and had the highest clinical score. The clinical scores of the ducks in the three deletion mutant groups, especially in the double deletion mutant APEC XMΔ*ryhB*Δ*neu* group, were significantly lower than those of the WT strain. This indicated that the deletion of *ryhB* and/or *neu* decreased the virulence of APEC XM. Interestingly, the virulence of APEC XMΔ*ryhB* was lower than that of APEC XMΔ*neu*. We supposed that RyhB could upregulate the virulence of APEC by not only regulating capsular synthesis but also regulating other virulence-related genes. The virulence of the three complemented mutants APEC XMΔ*ryhB*/p*ryhB*, APEC XMΔ*neu*/p*neu*, and APEC XMΔ*ryhB*Δ*neu*/p*ryhBneu* were similar to that of APEC XM. This indicated that the virulence of the three complemented mutants have been restored.

**Figure 6:**
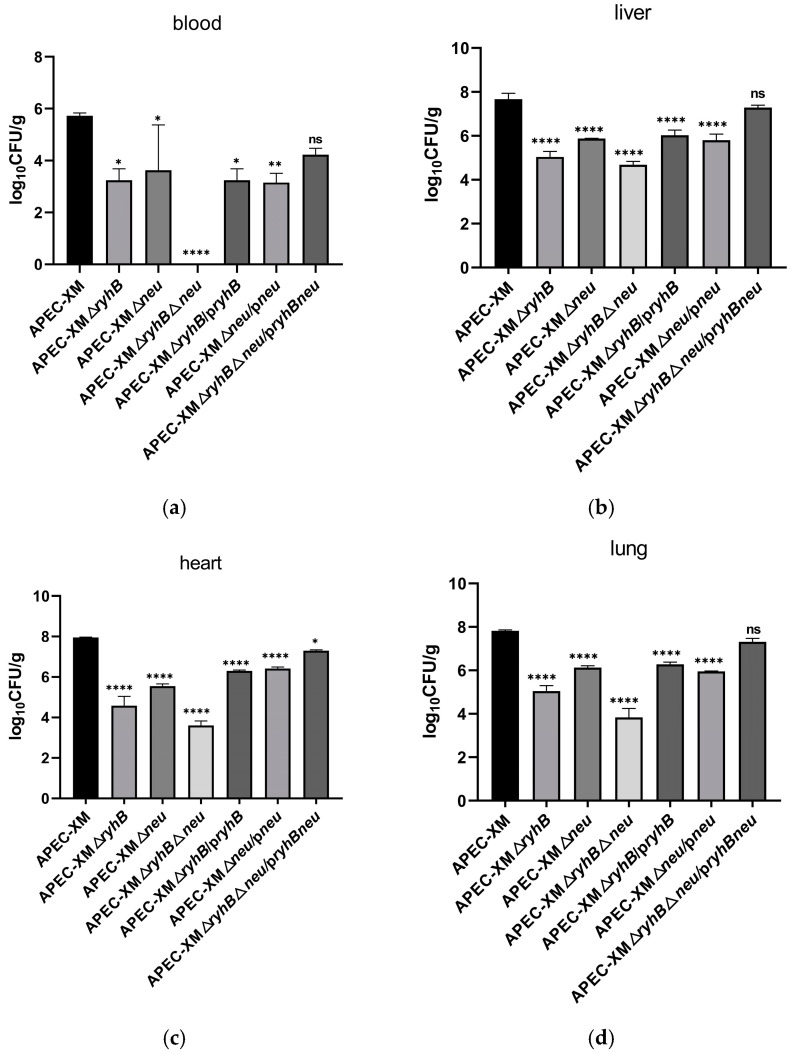
Bacterial loads in the (**a**) blood, (**b**) liver, (**c**) heart, and (**d**) lung of infected ducks at 20 h post-infection. The results were analyzed with one-way ANOVA by GraphPad Prism software and presented as the mean ± standard errors of the mean for three independent experiments. Tukey’s HSD test was used for multiple comparisons to determine differences between the APEC XM infection group and the mutant infection groups. Significant differences between APEC XM and all mutants are indicated by *p*-values. * *p* < 0.05; ** *p* < 0.01; **** *p* < 0.0001; ns: no significance, *p* > 0.05.

Compared with the APEC XM group, the bacterial loads in the blood decreased significantly in the three deletion mutant groups, especially in the APEC XMΔ*ryhB*Δ*neu* group at 20 h post-infection (Figure 6a). The bacterial loads in the liver, heart, and lung also decreased in the three deletion mutant groups compared with the APEC XM group but had no significant difference with that in the corresponding complemented mutant group (Figure 6b–d). In the APEC XMΔ*ryhB* group, the bacterial loads of all the above tissues were lower than those in the APEC XMΔ*neu* group.

LD50 assays were performed to assess the effect of RyhB and capsule on APEC XM virulence in ducks. The LD50 of the WT strain, APEC XMΔ*ryhB*, APEC XMΔ*neu*, and APEC XMΔ*ryhB*Δ*neu* was 7.11 × 10^5^, 7.55 × 10^7^, 5.00 × 10^7^, and 9.05 × 10^7^ colony forming unit (CFU), respectively (Table S1). Compared with the WT strain, the LD50 of deletion mutants APEC XMΔ*ryhB*, APEC XMΔ*neu*, and APEC XMΔ*ryhB*Δ*neu* significantly increased about 100-fold. This indicated that both RyhB and capsule were essential for APEC XM virulence.

## 3. Discussion

When ExPEC enters into the bloodstream environment, the bacteria need to adapt to the low-iron and -oxygen environment of the bloodstream and strong bactericidal effects of the serum. sRNA is critical for adapting to the stress environment. RyhB, an iron metabolism-related sRNA [15], can be induced in a low-oxygen serum environment and help APEC’s survival in the serum [4]. In our previous study, many virulence-related genes regulated by RyhB were identified by the transcriptome analysis. Among these genes, the most significant downregulated genes in the *ryhB* deletion mutant are the capsular cluster genes [4]. Capsule is critical for serum resistance. It is speculated that RyhB contributes to serum resistance and the pathogenicity of APEC mainly by directly upregulating the expression of capsule genes. So, it is essential to study the regulation mechanism of RyhB to capsular synthesis.

By now, a minority of trans-acting sRNAs such as RyhB upregulate target mRNA expression [29,30]. Some sRNA activates translation by disrupting an inhibitory secondary structure and releasing the ribosome binding site (RBS) [30,31,32]. In our study, the secondary structure prediction of *kpsF* and *kpsM* UTR presented a stem-loop structure, respectively, which may sequester the RBS and then block the translation initiation. An operon polarity suppressor (*ops*) sequence that located 28 bp upstream of *kpsM* can occlude the RBS and inhibit translation [33]. RfaH reads this sequence and facilitates the Region 2 of capsular gene cluster transcription [28]. Interestingly, our interaction site prediction showed that RyhB can interact with this ops sequence by incomplete base pairing. It is supposed that RyhB binds to the *ops* sequence, exposes the RBS, and activates translation. Further studies using the GFP-based reporter system proved that RyhB activated the 5′ UTR of *kpsF* and *kpsM* and promoted the translation of GFP. The production of capsular polysaccharide determination revealed that the content of polysaccharide in the APEC XMΔ*ryhB* mutant is higher than that in the APEC XMΔ*neu* mutant. This indicated that the deletion of *ryhB* reduced the synthesis of the capsule but did not result in losing the capsular synthesis ability. In other words, RyhB promotes capsular synthesis.

Capsule is a steric barrier outside the bacteria that is responsible for serum resistance *in vitro* [34] and virulence in animals [35]. K1 capsule can also enhance the survival ability of *E. coli* in brain cerebral microvascular endothelial cells [36]. In addition, K1 capsular polysialic acid binds to immunoglobulin-like lectin and escapes the killing of macrophages [37] and the degradation of lysosomes in macrophages, thus enhancing the bacteremia level and mortality of infected mice [38]. As a regulator, sRNA affects the pathogenicity of bacteria by regulating the expression of virulence-related factors. To study if RyhB regulates the pathogenicity of APEC by regulating capsular synthesis, the resistance to duck serum and macrophage phagocytosis was detected in the *ryhB* and/or *neuDBACES* deletion mutants. Compared with the APEC XMΔ*neu* mutant, the ability of resistance to macrophage phagocytosis in APEC XMΔ*ryhB* was attenuated, while in APEC XMΔ*ryhB*Δ*neu*, it was enhanced. It is supposed that the capsule plays a critical role in phagocytosis resistance. Besides the capsule, RyhB may regulate other factors related to phagocytosis resistance.

In this study, the pathogenicity of APEC was tested using natural host duck. The LD50 assay and clinical symptoms’ determination showed that the effect of RyhB on the virulence of APEC was greater than that of the capsule, although the production of the capsule in APEC XMΔ*neu* was less than that in APEC XMΔ*ryhB*. It is speculated that several virulence-related genes regulated by RyhB are involved in the pathogenicity of APEC. Moreover, our previous study showed that numerous virulence-related genes such as fimbrial genes and iron homeostasis genes were screened as positively regulated genes by RyhB from RNA-Seq data [4]. This indicated that RyhB was not only a virulence factor but also an important regulator. Although capsular cluster genes are the most significantly upregulated genes by RyhB, they are not the only target genes. Besides the upregulation of capsular cluster genes by RyhB, all virulence-related genes positively regulated by RyhB may jointly promote the pathogenicity of APEC.

## 4. Materials and Methods

### 4.1. Bacterial Strains, Plasmids, and Growth Conditions

The bacteria and plasmids used in this study are listed in Table 1. The characteristics of APEC XM strain (O2:K1), APEC XMΔ*ryhB*, and APEC XMΔ*ryhB*/p*ryhB* were described previously [4]. APEC XMΔ*neu*, APEC XMΔ*neu*/p*neu*, APEC XMΔ*ryhB*Δ*neu*, and APEC XMΔ*ryhB*Δ*neu*/p*ryhBneu* were constructed in this study. All bacteria were cultured in Luria–Bertani (LB) broth or on LB plates at 37 °C with agitation at 180 rpm. The mutants containing the temperature-sensitive plasmid pCP20 or pKD46 were grown in LB containing ampicillin (Amp, 100 μg/mL) (Sangon Biotech, Shanghai, China) or chloramphenicol (Cm, 34 μg/mL) (Sangon Biotech, Shanghai, China) when appropriate at 30 °C. Plasmids pKD3, pKD46, and pCP20 were used for the construction of a deletion mutant. Plasmids pBR322 and pACYC184 were used for constructing a complemented mutant.

### 4.2. Quantitative Real-Time PCR

APEC XM and the APEC XMΔ*ryhB* mutant were grown in LB medium at 37 °C until the exponential phase. Then, the bacteria were collected by centrifugation, washed twice with PBS to remove the LB medium, resuspended in duck serum, and incubated at 37 °C under low-oxygen (2.5%) conditions for 2 h. After the above treatment, the bacteria were collected, and qRT-PCR was carried out as described previously [4]. Briefly, all of the primers used to amplify *kpsF*, *kpsM*, *kpsU*, *neuC*, and *neuD* are shown in Table S2. Total RNA was extracted using TRIzol reagent (Invitrogen, Carlsbad, CA, USA) from bacteria incubated in duck serum. qRT-PCR was performed on an ABI7500 instrument (Applied Biosystems, Carlsbad, CA, USA) using the SYBR Premix Ex Taq II (Takara, Tokyo, Japan). The relative mRNA expression of each gene was evaluated using the 2^−∆∆Ct^ method and normalized to the endogenous reference genes *gapA*. Assays were performed in triplicate.

### 4.3. Prediction of Interaction Sites and the Secondary Structure of Target mRNA

The interaction sites between RyhB and target mRNA were predicted as previously described [40,41,42]. Briefly, a 365-nucleotide sequence, which contained a 165 nt 5′ UTR of the *kpsF* and the first 150 bases of the *kpsF* coding sequence, and the whole sequences of *ryhB* were submitted to the IntaRNA website for *kpsF*-*ryhB* interaction site prediction. For *kpsM*-*ryhB* interaction site prediction, a 300-nucleotide sequence, which contained a 150 nt 5′ UTR of the *kpsM* and the first 150 bases of the *kpsM* coding sequence, and the whole *ryhB* sequences, were submitted to the website. The secondary structure of candidate target mRNAs with their 5′ UTR was predicted by the RNAstructure module of the CLC Main Workbench (5.5) [40,43].

### 4.4. Validation of Interactions Between RyhB and Targets by a GFP-Based Reporter System

The interaction of RyhB and target mRNA was detected using the GFP-based reporter system as previously described [40,44]. Briefly, the 5′ UTR sequence of *kpsF* and *kpsM* were separately cloned upstream of *gfp* gene’s initiation codon in the GFP expression plasmid pXG-10SF to construct fusion expression plasmids 5′ UTR *kpsF*-pXG-10SF and 5′ UTR *kpsM*-pXG-10SF, respectively. The *ryhB* sequence was cloned into sRNA expression plasmid pJV-300 to generate plasmid *ryhB*-pJV-300. The primers used for cloning and vector construction are provided in Table S3. The plasmids 5′ UTR *kpsF*-pXG-10SF (or 5′ UTR *kpsM*-pXG-10SF) and *ryhB*-pJV-300 were transformed to *E. coli* strain Top10 to the co-expression of GFP fusions and RyhB. The fluorescence of *E. coli* strain Top10 harboring a *gfp* fusion plasmid and a RyhB expression plasmid was measured as described previously [40].

### 4.5. Construction of the Deletion Mutants and the Complemented Mutants

All the deletion mutants were constructed using the λ-Red-mediated recombination system, as described previously [39,40]. The primers used for gene cloning and mutant construction are given in Table S4. Primers *neu-D-F* and *neu-D-R* containing the homologous region of the *neu* sequence were used to amplify the chloramphenicol (Cm) cassette from plasmid pKD3. The allelic replacement of the whole *neuDBACES* sequence by the Cm cassette and the excision of the Cm cassette was verified by PCR and DNA sequencing. The double deletion mutant APEC XMΔ*ryhB*Δ*neu* was generated by operating the *neu* deletion process in mutant APEC XMΔ*ryhB*. The complemented mutant was generated by cloning the full-length *neuDBACES* sequence into plasmid pACYC184 or *ryhB* sequence into pBR322, which was transformed to the corresponding single deletion mutant. Both plasmid pACYC-*neu* and pBR-*ryhB* were transformed to the double deletion mutant to construct the double complemented mutant.

### 4.6. Extraction and Quantification of Capsules When APEC Resisted to Duck Serum

APEC XM, deletion mutants APEC XMΔ*ryhB*, APEC XMΔ*neu*, and APEC XMΔ*ryhB*Δ*neu*, and complemented mutants APEC XMΔ*ryhB*/p*ryhB*, APEC XMΔ*neu*/p*neu*, and APEC XMΔ*ryhB*Δ*neu*/p*ryhBneu* were cultured in LB medium to log phase. The bacterial cultures were transferred to duck serum at ratio of 1:100 and grown in low-oxygen (2.5%) for 8 h. Then, capsules of all of the above strains were extracted and quantified using the method described previously [8]. The content of the capsules was determined by absorbance at 520 nm.

### 4.7. Survival of APEC in Duck Serum

The assay of APEC survival in serum was performed as described previously [4]. The whole blood duck serum was prepared from 8-week-old pathogen-free ducks. APEC XM, deletion mutants APEC XMΔ*ryhB*, APEC XMΔ*neu*, and APEC XMΔ*ryhB*Δ*neu*, and complemented mutants APEC XMΔ*ryhB*/p*ryhB*, APEC XMΔ*neu*/p*neu*, and APEC XMΔ*ryhB*Δ*neu*/p*ryhBneu* were cultured in LB medium at 37 °C to exponential phase. Then, the bacteria were centrifuged, washed twice with PBS, resuspended in duck serum with a 1:20 dilution of the original bacteria amount, and incubated at 37 °C under low-oxygen (2.5%) conditions for 0.5 h, 1 h, and 2 h, respectively. Assays were performed in triplicate. The survival rate of bacteria at different time points was calculated using the ratio of bacteria number at the above time point to the number at 0 h for each strain.

### 4.8. Determination of Resistance to Phagocytosis

The mice macrophage cells RAW264.7 were cultured in Dulbecco’s Modified Eagle Medium (DMEM; HyClone, Logan, UT, USA) containing 10% heat-inactivated fetal bovine serum (FBS; Gibco, Carlsbad, CA, USA) at 37 °C in an atmosphere of 5% CO_2_. Bacteria were grown in LB medium for 2 h to log phase, washed with DMEM medium, and adjusted to 1 × 10^7^ CFU/200 µL. Then, the above bacteria were incubated on a monolayer of 1 × 10^5^ RAW264.7 cells at a multiplicity of infection (MOI) of 100 at 37 °C in 48-well culture plates for 1 h. After 1 h of infection, the solution was removed. The cells were gently washed twice with PBS and treated with DMEM containing 50 µg/mL gentamycin for 1 h to kill the bacteria outside the cells. Then, the cell monolayers were washed twice with PBS and lysed with 1% Triton X-100 (Solarbio, Beijing, China) for 30 min. The lysates were serially diluted and plated onto LB agar plates. The exact number of bacteria phagocytosed by RAW264.7 cells was determined using the CFU count on LB plates. The ratio of phagocytosis was determined by calculating the bacteria number inside the macrophage to the number of initial bacteria that were incubated with the cells.

### 4.9. Animal Infections

All animal experiments were approved by the Institutional Animal Care and Use Committee of the Yangzhou University Animal Experiments Ethics Committee and followed the National Institute of Health guidelines for the ethical use of animals in China (code No. 202302121). To evaluate the effect of RyhB and capsule on APEC virulence, forty 7-day-old ducks were randomly separated into one control group and seven bacterial infection groups (APEC XM, APEC XMΔ*ryhB*, APEC XMΔ*neu*, APEC XMΔ*ryhB*Δ*neu*, APEC XMΔ*ryhB*/p*ryhB*, APEC XMΔ*neu*/p*neu*, and APEC XMΔ*ryhB*Δ*neu*/p*ryhBneu*), with five ducks in each group. The ducks were inoculated intraperitoneally with a dose of 1 × 10^7^ CFU bacteria in 200 µL PBS or an equal volume of PBS. The clinical symptoms of the ducks were observed 20 h post-infection. The health status of the ducks was assessed by a clinical score (0, behavioral normality; 1, slight depression; 2, moderate depression, rare spontaneous movements, no diarrhea; 3, severe depression, diarrhea, anorexia, and opisthotonus; 4, dead). When the clinical score of a duck reached 3, it was sacrificed for ethical reasons. None of the animals died spontaneously. Ducks were euthanized at 20 h post-infection. Blood, hearts, livers, and lungs were immediately collected aseptically for tissue bacterial load assessment.

### 4.10. Determination of Bacterial Loadings in the Tissues of Duck

The bacterial loadings in the tissues were determined as described previously [45]. The tissue samples, including hearts, livers, and lungs, were homogenized with sterile pre-cool PBS. The homogenates were diluted serially tenfold, plated on MacConkey plates, and cultured at 37 °C for determining CFUs. The bacterial loadings were calculated by CFUs per gram of tissues or per microliter of blood.

### 4.11. LD50 Assay

The lethal dose 50% (LD50) was calculated 10 days post-infection using the method described previously [46]. Briefly, 7-day-old ducks were randomly divided into four infection groups and one control group (n = 20). APEC XM and deletion mutants APEC XMΔ*ryhB*, APEC XMΔ*neu*, and APEC XMΔ*ryhB*Δ*neu* were cultured to log phase with an OD600 of 1, harvested by centrifugation, washed, and resuspended to 1 × 10^5^ CFU/mL, 1 × 10^6^ CFU/mL, 1 × 10^7^ CFU/mL, and 1 × 10^8^ CFU/mL gradient suspensions in sterile PBS. The infection groups of the ducks were inoculated with 200 μL of the above gradient suspensions of four strains separately, while the control group was inoculated with 200 μL of PBS by subcutaneous injection. The LD50 was calculated 10 days post-infection using the method described previously [47].

### 4.12. Statistical Analysis

Statistical analysis was performed by GraphPad Prism 9.5 software (GraphPad Software, San Diego, CA, USA). The data of qRT-PCR and relative fluorescence determination were analyzed using an unpaired Student’s *t*-test, while other data were analyzed by one-way analysis of variance (ANOVA). Tukey’s HSD (Honestly Significant Difference) test was used for multiple comparisons to determine differences between the WT strain group and the mutant group. All data are represented as mean ± standard deviations (SDs) from triplicate independent experiments. Significant differences are indicated by *p*-values. *p*-value ≤ 0.05 is considered to be statistically significant.

## 5. Conclusions

RyhB in APEC directly upregulates the expression of the capsular genes cluster. RyhB contributes to resistance to serum and macrophage phagocytosis and pathogenicity to ducks partly by activating capsular synthesis. This study enriches our understanding of the pathogenic mechanisms of APEC involving sRNAs. A novel strategy was probably provided to control colibacillosis by developing an sRNA-mediated product such as attenuated vaccines.

## Figures and Tables

**Table 1:** Bacteria and plasmids used in this study.

Strain or Plasmid	Characteristic or Function	References
APEC XM	Virulent strain of APEC, isolated from the brain of duck	[4]
APEC XMΔ*ryhB*	Deletion mutant of *ryhB* with APEC XM background	[4]
APEC XMΔ*ryhB*/p*ryhB*	APEC XM Δ*ryhB* carrying the vector pBR-*ryhB*, Amp^r^	[4]
APEC XMΔ*neu*	Deletion mutant of *neuDBACES* with APEC XM background	This study
APEC XMΔ*neu*/p*neu*	APEC XM Δ*neu* carrying the vector pACYC184-*neu*, Cm^r^	This study
APEC XMΔ*ryhB*Δ*neu*	Deletion mutant of *ryhB* and *neuDBACES*	This study
APEC XMΔ*ryhB*Δ*neu*/p*ryhBneu*	APEC XMΔ*ryhB*Δ*neu* carrying the vector pBR-*ryhB* and pACYC-*neu*	This study
pKD46	Amp^r^, λ-red recombinase expression	[39]
pKD3	Cm^r^, Cm cassette template	[39]
pCP20	Amp^r^, Cm^r^, Flp recombinase expression	[39]
pBR-*ryhB*	Amp^r^, pBR322 carrying the full *ryhB* gene sequence	[4]
pACYC184-*neu*	Cm^r^, pACYC184 carrying the full *neuDBACES* sequence	This study
pJV-300	Amp^r^, sRNA cloning vector	[40]
pXG-10SF	Cm^r^, target gene cloning vector with GFP	[40]

## Data Availability

The datasets used and analyzed during the current study are available from the corresponding author on reasonable request.

## Supplementary Materials

The following supporting information can be downloaded at: https://www.mdpi.com/article/10.3390/ijms26073062/s1.

## Abbreviations

The following abbreviations are used in this manuscript:

APEC	Avian pathogenic *Escherichia coli*
ExPEC	Extraintestinal pathogenic *Escherichia coli*
sRNA	small non-coding RNA
H-NS	histone-like nucleoid structuring protein
IHF	Integration Host Factor
*kps*	capsule gene clusters
qRT-PCR	quantitative real-time PCR
WT	wild type
UTR	untranslated region
SD	Shine–Dalgarno
*ops*	operon polarity suppressor
LB	Luria–Bertani
CFU	colony forming unit
LD50	lethal dose 50%
RBS	ribosome binding site
DMEM	Dulbecco’s Modified Eagle Medium
FBS	fetal bovine serum
MOI	multiplicity of infection
