# Supplementary material for: RyhB Regulates Capsular Synthesis for Serum Resistance and Virulence of Avian Pathogenic Escherichia coli
Source: Int J Mol Sci. 2025 Mar 27;26(7):3062. doi: 10.3390/ijms26073062 (PMC11988350; doi:10.3390/ijms26073062)
Supplement: Supplementary file 1 [file ijms-26-03062-s001.zip › ijms-3461051-supplementary.pdf]

Supplementary materials

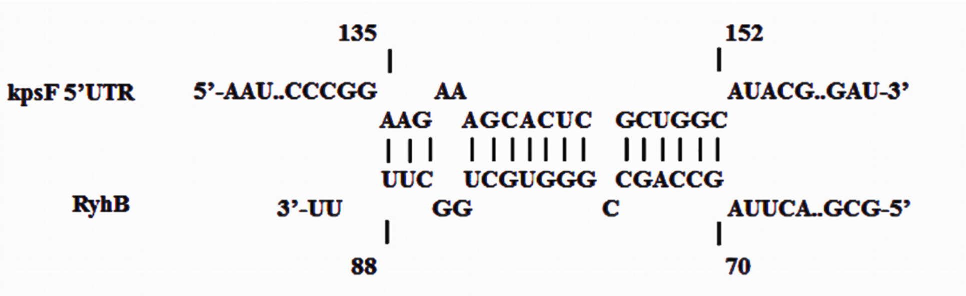

(a)

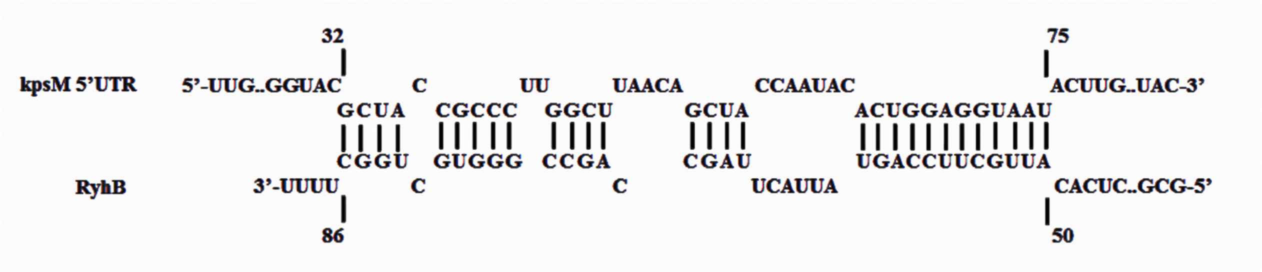

(b)

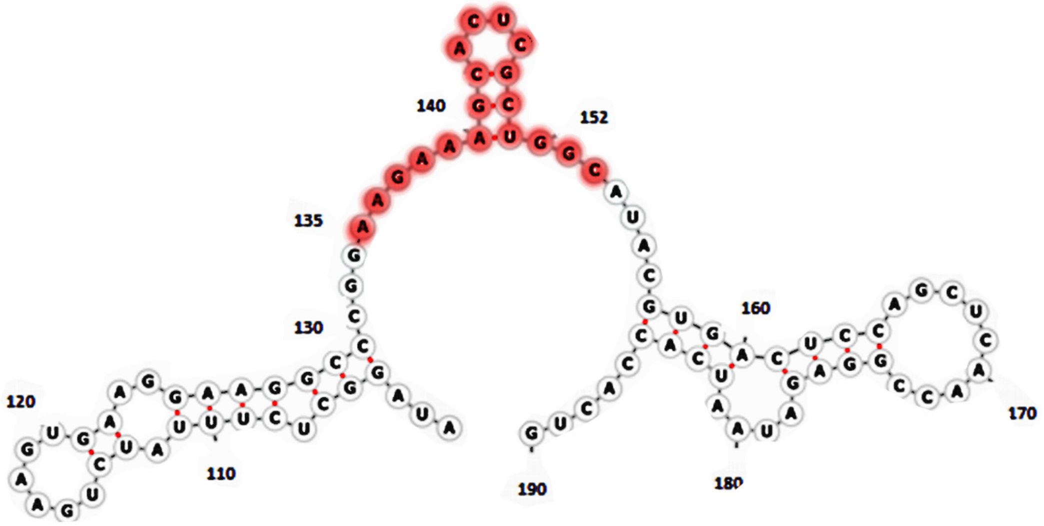

(c)

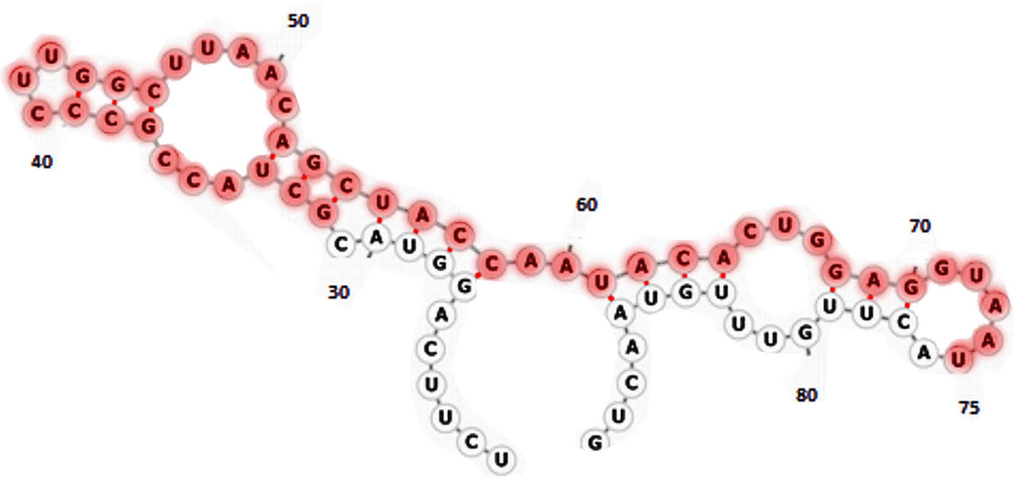

(d)

Figure S1. Prediction of interaction sites and the secondary structure of target mRNA. (**a**) Interaction sites prediction between RyhB and the 5’UTR of *kpsF*; (**b**) Interaction sites prediction between RyhB and the 5’UTR of *kpsM*; (**c**) The secondary structure prediction of the 5’UTR and the first 150 bp mRNA of *kpsF*. The 5’UTR of *kpsF* (nt 135-152) that form a stem-loop is marked in red; (**d**) The secondary structure prediction of the 5’UTR and the first 150 bp mRNA of *kpsM*. The 5’UTR of *kpsM* (nt 32-75) that form a long stem-loop is marked in red.

Table S1. LD50 assays of APEC XM and deletion mutants to ducks.

		Dead number/Total number			
**Strain**	**1×10^5**	**1×10^6**	**1×10^7**	**1×10^8**	**LD_{50}**
APEC XM	1/5	3/5	5/5	5/5	7.111×10^5
APEC XM Δ*ryhB*	0/5	0/5	2/5	3/5	7.551×10^7
APEC XM Δ*neu*	0/5	1/5	1/5	5/5	5.000×10^7
APEC XMΔ*ryhB*Δ*neu*	0/5	0/5	1/5	3/5	9.050×10^7

Table S2. The sequence of primers for qRT-PCR.

Primer	Sequences (5′-3′)
kpsF-RT -F	ATGGCGATCTGGGCATGATT
kpsF-RT -R	CATTTTTCGCCAGCGTGGAA
kpsU-RT -F	GCCAAGCACGGTAAAAGTGG
kpsU-RT -R	TGCTCCAGTGATTCTGCCTG
kpsM-RT -F	CAGTCCCTGGTTCGCTTCAA
kpsM-RT -R	CTCCGCGCATTTGCTGATAC
neuC-RT -F	ACCAGAGGAGGAGTTCCCAA

neuC-RT -R	TTGGCAGTAACGCTGACACT
neuD-RT -F	GCTTCCTAGTTGATGGTTTGCC
neuD-RT -R	TTTGGTGCGGGTGGTTTTTC
GapA-RT-F	CGTTAAAGGCGCTAACTTCG
GapA-RT-R	ACGGTGGTCATCAGACCTTC

Table S3. The sequence of primers for vector construction.

Primer	Sequences (5′-3′)
pXG-10SF-Fwd	GCTGCTGGTTCTGGCGAATTC
pXG-l0SF-Rev	ATGCATGTGCTCAGTATCTCTATCAC
pXG-l0SF-seq-Fwd	CGCCAGATATCGACGTCT
pXG-0SF-seq-Rev	CCTCTCCACGGACAGAAA
KpsF-pXG-10SF-Fwd	GTGATAGAGATACTGAGCACATGCAT<br>CCTTGTTCATAATGTAGGGGTG
KpsF-pXG-10SF-Red	GAATTCGCCAGAACCAGCAGC<br>ATCGATAGTACTGCTCTGGTC
KpsM-pXG-10SF-Fwd	GTGATAGAGATACTGAGCACATGCAT<br>GTGATCCTAATCTCTTCAGGTACGCTA
KpsM-pXG-10SF-Red	GAATTCGCCAGAACCAGCAGC<br>GAAATTACCAGAAGACCGAA

Table S4. Primers used for mutant construction.

Primer	Sequences (5′-3′)
neu-D-F	GGTTATCATCAATCATATCCAATATTAGGTAATGAT
	ATTGCAGACATCGATGTGTAGGCTGGAGCTGCTTCG
neu-D-R	GTAAGATAGAAATTAACGAATCCGCAAATAATGT
	ATGTTCAATCAAATACCATATGAATATCCTCCTTAG
neu-I-F	TGGTGCGGGTGGTTTTTCAAA
neu-I-R	TTAGGCTCTTTAGGGTGAGGTTT
neu-hb-F	ATTTCTATGCGCACCCGTTCACATAGTGAACGAGCTTT
neu-hb-R	AGCCTGATAAAAACGGTTAGCGCAGCTTTTATTACTCCCCAAG
neuIP-F	ATCGTGCAAAGAGGGAGAT
neuIP-R	CAAAGAGGGGCAAGGTAAA
ryhBIP-F	GGCGCAGTTAATGACCATG
ryhBIP-R	GTAATACACCTCCGGCTGGG
pACYC-F	GCGCTAACCGTTTTTATCAGGCT
pACYC-R	GAACGGGTGCGCATAGAAAT
